# Heavy Metal Toxicity in Clinical and Environmental Health: Sources, Mechanisms, Diagnostics, and Evidence-Based Management of Mercury, Lead, Cadmium, and Arsenic

**DOI:** 10.3390/ijms27083513

**Published:** 2026-04-14

**Authors:** Dib Chakif, Julien Furrer

**Affiliations:** Department of Chemistry, Biochemistry and Pharmaceutical Sciences, University of Bern, 3012 Bern, Switzerland; julien.furrer@unibe.ch

**Keywords:** heavy metals, mercury, lead, cadmium, arsenic, metal toxicity, environmental exposure, bioaccumulation, neurotoxicity, chelation therapy, risk assessment

## Abstract

Heavy metals including mercury (Hg), lead (Pb), cadmium (Cd), and arsenic (As) remain significant global toxins due to their environmental persistence, widespread anthropogenic release, and serious biological effects. This review consolidates current understanding of their natural and industrial sources, environmental cycling, human exposure routes, and population-level vulnerabilities. It covers their toxicokinetics and toxicodynamics, emphasizing species-specific absorption, distribution, and injury mechanisms, including oxidative stress, thiol binding, mitochondrial dysfunction, endocrine disruption, and cancer risk. Clinical signs range from subtle neurocognitive impairment and kidney damage to severe acute poisoning. The review evaluates evidence-based approaches to risk assessment and biomonitoring, such as blood, urine, hair, and speciation tests, noting issues, including unvalidated provoked testing. Treatment focuses on removing exposure, providing nutritional support, and offering supportive care, with chelation therapy reserved for specific cases. It explains the chemistry, pharmacology, and roles of chelating agents—ALA, DMSA, DMPS, Cys, GSH, and physiologic thiols, comparing their effectiveness, limitations, and costs for various metals. Emerging therapies, precision toxicology, and public health strategies are discussed within a prevention-focused context. Unlike prior reviews focused primarily on toxic mechanisms or isolated clinical management, this review integrates mechanistic toxicology, biomarker interpretation and speciation, evidence-based clinical care, and ethical, cost-conscious decision-making within a single translational framework. This narrative review synthesizes foundational and contemporary literature published through 2025, with particular emphasis on studies published since 2000 that inform toxicokinetics, biomarker interpretation, diagnostics, clinical management, and prevention.

## 1. Introduction

**Figure 1:**
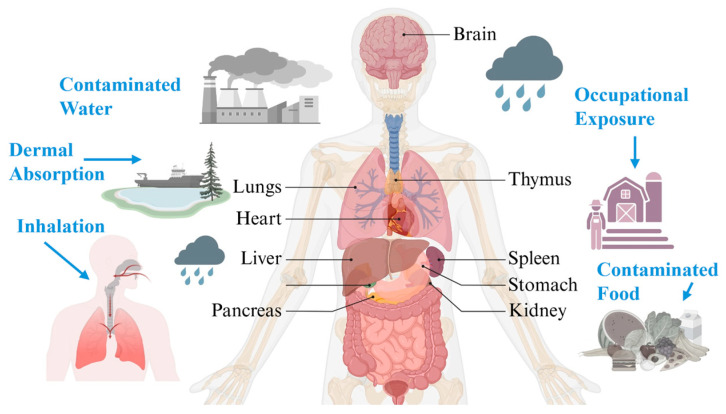
Exposure pathways and organ distribution of toxic contaminants in the human body.

Heavy metals, including mercury (Hg), lead (Pb), cadmium (Cd), and arsenic (As), are among the most intensively studied toxicants in environmental medicine and clinical toxicology. These elements and metalloids are naturally occurring substances distributed widely in the earth’s crust throughout ecological cycles, and some have been classified as one of the most toxic elements approved by the World Health Organization (WHO) as chemicals of major public health concern (see Figure 1) [1,2]. They originate mainly from natural sources, including volcanic activity, erosion, weathering of mineral deposits, and biological cycling through ecosystems [3,4,5].

During volcanic eruptions, a variety of metals are released into the atmosphere, including lead, mercury, and cadmium, which can remain in the atmosphere for extended periods, depending on the type and scale of the eruption [6]. Elemental mercury vapor is efficiently absorbed via the lungs and penetrates the central nervous system; inorganic salts concentrate in the kidney; organic methylmercury (MeHg), formed in aquatic food webs, is absorbed from the diet and avidly enters the fetal and adult brain.

Lead’s chemistry enables it to mimic calcium and bind to sulfhydryl and carboxyl groups, disrupting neurotransmission, heme synthesis, and bone metabolism [7].

Cadmium binds to metallothionein and progressively accumulates in the renal cortex, with biological half-lives measured in decades [8].

Arsenic occurs as trivalent arsenite and pentavalent arsenate, which undergo hepatic methylation to monomethylarsonic acid (MMA) and dimethylarsinic acid (DMA); chronic exposure—often via groundwater or rice—has multisystem consequences including dermatologic, vascular, metabolic, and oncologic outcomes [9].

Clinicians continue to encounter patients with exposures ranging from dramatic acute poisonings to subtle, chronic, low-level burdens that still confer measurable risk. Several previous reviews have summarized the environmental occurrence and toxic mechanisms of heavy metals, including broad overviews of arsenic, cadmium, chromium, mercury, and lead, as well as more focused mechanistic reviews [10,11]. However, these reviews do not fully integrate speciation-informed diagnosis, bedside decision pathways, stewardship of chelation, and cost-conscious prevention into a unified clinical and public health model.

What distinguishes the present review from prior reviews is its integrative, decision-oriented framework spanning the full pathway from exposure biology to bedside and public-health action. Rather than discussing heavy metal toxicity only in terms of toxic mechanisms or only as a clinical management problem, this review brings together mechanistic toxicology, species-specific biomarker interpretation and speciation, evidence-based clinical management, and the ethical and economic realities that shape real-world care. It also synthesizes more recent evidence on comparative effectiveness, stewardship of chelation, implementation barriers, equity, and emerging precision-toxicology approaches. By linking mercury, lead, cadmium, and arsenic within a single framework that integrates diagnosis, prevention, treatment, and policy, this review aims to offer a more practical and conceptually updated model for clinicians, toxicologists, and public health practitioners than reviews focused on isolated metals or narrower toxicological endpoints.

## 2. Sources and Environmental Fate

Mercury, lead, cadmium, and arsenic can also be naturally bound to other mineral compounds (see Table 1). For instance, Hg can bind to sulfur (e.g., cinnabar, HgS) and selenium (e.g., tiemannite, HgSe), as well as to other metals such as gold (Au) and silver (Ag) [12]. Pb can also bind to sulfur (e.g., galena, PbS) and others, such as sulfates (e.g., anglesite, PbSO_4_) and carbonates (e.g., cerussite, PbCO_3_), and is frequently associated with zinc (Zn) ores (e.g., sphalerite, ZnS), and is commonly associated with Pb, Iron (Fe), and Copper (Cu) ores [13,14].

Mercury is a globally distributed pollutant with important natural, anthropogenic, and re-emitted sources. Current estimates indicate that anthropogenic emissions to air are on the order of 2200 metric tons per year, whereas total global atmospheric mercury inputs are substantially higher when natural sources and re-emission from land and ocean reservoirs are included. Coal combustion is an important source, but artisanal and small-scale gold mining is generally considered the largest single anthropogenic source of mercury releases globally, and coal combustion accounts for roughly one-fifth rather than most of global anthropogenic atmospheric emissions. Mercury in the environment occurs as elemental, inorganic, and organic species, and its environmental behavior depends strongly on chemical form [18,19].

Elemental mercury (Hg^0^) has been used in thermometers, switches, relays, some lighting products, dental amalgams, and other industrial or legacy applications. It is the only metal that is liquid at room temperature and can enter the atmosphere in vapor form. Organic mercury is dominated environmentally by methylmercury (CH_3_Hg^+^), which bioaccumulates in aquatic food webs and is a major form in fish. Ethylmercury is relevant primarily as a metabolite of thimerosal, a preservative still used in some multi-dose vaccine vials, particularly certain influenza vaccines [20] In some cases, Hg is bound to airborne particles, which can be transported long before settling on the Earth’s surface. The interest in mercury toxicity has increased in modern science. However, it has been known as a neurotoxic substance for centuries, yet there remains controversy on the mechanism of interaction with biochemical processes.

Once elemental Hg is released into the air, it is transported over vast distances by wind patterns, where it undergoes complex atmospheric chemical reactions, such as oxidation, photo-oxidation, and particulate binding [15]. Oxidation of Hg occurs by atmospheric compounds such as ozone (O_3_) and halogens (e.g., Cl). The process leads to the formation of oxidized Hg (Hg^2+^), which is readily removed from the atmosphere due to its water solubility. The photo-oxidation of Hg can be promoted by ultraviolet light, increasing its potential for deposition in aquatic ecosystems [21].

Moreover, some oxidized Hg can bind to particulate matter, which can be later removed by gravity or precipitation [22]. Once Hg is transformed, it is removed from the atmosphere through wet and dry deposition. Wet deposition promotes Hg entry into ecosystems via surface waters or onto the land through rain, snow, fog, etc. [23]. Dry deposition promotes Hg to settle onto surfaces such as soil, water bodies, and vegetation without precipitation. However, Hg does not remain stationary in the environment due to Hg re-emission processes. Hg deposited on soil and water can be re-emitted into the atmosphere under certain conditions, such as high temperatures or changes in the chemical environment. When Hg is present in water bodies or soil, microbial activity or temperature changes can volatilize Hg back into the air as elemental Hg through evaporation [24]. Mercury deposited on plants can also be re-emitted in the atmosphere through foliar uptake and evapotranspiration [25]. However, anthropogenic activities have significantly altered the presence and concentration of these metals in the environment [6].

Lead (Pb) is known for its unique physicochemical properties, such as malleability, low electrical conductivity, softness, and slow dissolution in water. It has been used for centuries in many applications, including the manufacture of drinking water pipes and as an additive to gasoline [7]. Leaded petrol was one of the most significant sources of atmospheric lead in the 20th century. Lead compounds were added to gasoline as an anti-knock agent and were also incorporated into paint [26]. In developed countries, the use of lead is limited, but it remains in lead-containing products, such as gasoline, pigments, ceramics, and batteries [7]. Despite its beneficial contribution in many applications, lead is highly toxic and is one of the most widespread environmental health hazards. Because of its widespread use in many applications, it can enter the environment primarily through anthropogenic activities such as land application, mining, and smelting, via the recycling of waste materials and the combustion of fossil fuels [27]. Lead is also found in small quantities in cigarette smoke. The tobacco plant can accumulate lead from the soil, and when burned, small amounts of lead are released into the atmosphere [28].

Pb is introduced into the atmosphere during the weathering of rocks and minerals, where the natural breakdown of rocks and minerals takes place through physical and chemical weathering processes. Over time, trace amounts of lead are released into the atmosphere and become airborne [29]. Nowadays, Pb emissions exceed 100,000 metric tons per year from both natural and human activities. Though this is a relatively minor source compared to industrial activities, it contributes to local lead contamination, particularly in areas with high smoking rates [28]. In countries where lead-based paint is still in use, sanding, scraping, or burning these paints can release lead particles into the air. This is particularly common in older buildings and homes. Recycling lead-containing materials, such as lead-acid batteries or old lead-based products, can also contribute to atmospheric lead. Improper handling or processing in certain recycling facilities can release lead particles [30,31,32].

Coal contains trace amounts of cadmium, and when coal is burned for energy production, cadmium is released into the atmosphere as part of the emissions. Coal-fired power plants are significant sources of atmospheric cadmium [25]. Cadmium is also commonly found in ores that contain zinc, lead, and copper. Smelting and refining these metals often release cadmium as a byproduct. Smelters are significant sources of cadmium emissions, especially in regions with prevalent zinc production [16]. Historically, cadmium was used in paints as a pigment, particularly in yellow, red, and orange hues. Although its use has declined significantly due to health concerns, cadmium can still be found in older paints. Sanding or scraping old painted surfaces can release cadmium into the air, contributing to its presence in indoor and outdoor environments [33]. The extraction of metals such as zinc, lead, and copper often releases cadmium into the environment as a byproduct. During mining and ore processing, cadmium can be emitted into the atmosphere, water, and soil. Mining operations in regions with high cadmium concentrations in ores are a significant source of environmental cadmium pollution [34]. Cadmium is used in the production of rechargeable batteries. The production and disposal of these batteries can result in cadmium emissions, mainly when they are improperly disposed of or recycled. Cement manufacturing can release trace amounts of cadmium into the atmosphere, primarily when raw materials containing cadmium are used in production. This source is considered minor compared to others, but it can still contribute to localized cadmium pollution, particularly in areas with high cement production [16]. These activities release cadmium into the air, water, and soil. The primary industrial sources include coal combustion, metal smelting and refining, paint pigment production, and mining operations [18,30]. Anthropogenic activities are the primary contributors to cadmium pollution in the environment, with cadmium emissions exceeding 3000 metric tons annually [35].

Arsenic is widely present in the Earth’s crust and commonly co-occurs with iron (Fe) and sulfur (S) in sulfide ores, as well as with precious and base metals [36]. In nature, it is frequently bound within minerals such as arsenopyrite (FeAsS), realgar (As_4_S_4_), orpiment (As_2_S_3_), and iron arsenates like scorodite (FeAsO_4_·2H_2_O); it also occurs in copper-arsenic sulfides (e.g., enargite and tennantite) and is often associated with gold (Au) and copper (Cu) deposits. These associations reflect hydrothermal and metamorphic processes that concentrate arsenic in polymetallic ore bodies. Globally, atmospheric arsenic emissions arise from both natural and anthropogenic sources [17]. Best current budgets indicate that anthropogenic emissions to the atmosphere (~17–38 × 10^9^ g As·yr^−1^) are roughly double the natural background (~10–25 × 10^9^ g As·yr^−1^), yielding a total on the order of 27–63 kilotons per year. Major natural contributors include volcanic/geothermal activity and wind-blown dust, whereas anthropogenic releases are dominated by coal combustion, non-ferrous metal smelting/roasting, and industrial processes.

## 3. Human Exposure and Risk Assessment

Human exposure to mercury (Hg), lead (Pb), cadmium (Cd), and arsenic (As) occurs through distinct but overlapping dietary, environmental, occupational, and household pathways [37]. Unlike essential trace elements such as iron, zinc, copper, manganese, molybdenum, cobalt, and selenium, these toxic metals and metalloids have no known beneficial physiologic role at environmentally relevant exposure levels. Their health effects depend on chemical form, dose, route, timing, co-exposures, and host susceptibility (see Table 2) [34].

For the general population, the main source of mercury exposure is dietary intake of methylmercury, primarily through fish and seafood. Methylmercury is the mercury species of greatest concern in food because it bioaccumulates in aquatic food webs and readily crosses both the placenta and the blood–brain barrier. Additional mercury exposure may occur in occupational settings, through accidental elemental mercury spills, and from some mercury-containing products. Dental amalgam contributes low-level mercury vapor exposure, but current evidence does not support routine removal of intact amalgam fillings in the general population [38]. The health effects of mercury depend strongly on speciation. Elemental mercury vapor is absorbed efficiently through the lungs and primarily affects the nervous system; inorganic mercury salts are more closely associated with renal toxicity; and methylmercury is particularly neurotoxic, especially during fetal and early-life development. Prenatal and early childhood methylmercury exposure has been associated with impaired cognition, attention, motor performance, and other neurodevelopmental outcomes [15].

Various studies have described molecular and cellular effects of organic Hg in the nervous system, which found that Hg^2+^ may play a crucial role after exposure to CH_3_Hg^+^ and CH_3_CH_2_Hg^+,^ and have suggested that the existence of Hg^2+^ in neurons results from the breakdown of organic Hg in glial cells, and that the levels of Hg^2+^ were higher after exposure to CH_3_CH_2_Hg^+^ [39]. Methylmercury exposure, particularly during pregnancy, infancy, and childhood, is associated with developmental and neurological effects [40]. Previous studies suggest that exposure to low levels of mercury may cause an increase in the risk of cardiovascular diseases, including hypertension and heart disease [41,42]. The exposure may also affect the immune system, leading to autoimmunity and increased susceptibility to infections by altering immune responses and causing inflammatory reactions [42]. All forms of mercury are one of the leading causes of adverse effects, including respiratory, immune, dermatologic, renal, reproductive, and developmental sequelae [40]. Researchers have also suspected that Hg is involved in the etiology of neurodegenerative diseases such as Alzheimer’s disease (AD), Amyotrophic Lateral Sclerosis (ALS), Multiple Sclerosis (MS), Parkinson’s Disease (PD), or autism spectrum disorder (ASD) [40].

Lead is a potent neurotoxin that poses significant risks to the nervous system, with even minimal exposure having detrimental effects [43]. Although less common than ingestion or inhalation, dermal exposure to lead can occur in specific occupational settings where workers handle lead-containing materials. Despite the widely recognized health hazards, lead exposure remains a significant issue, especially for high-risk groups like children, pregnant women, and individuals employed in specific industries. The effects of lead exposure are well-established, and even low levels of exposure can cause a range of adverse health outcomes, including developmental delays, cognitive impairments, and neurological damage [27]. As lead-based paint deteriorates, it produces lead dust, which can settle on surfaces and become a source of exposure when children play on floors, touch contaminated surfaces, or put their hands in their mouths [25,44].

Children are particularly at risk, as low levels of lead exposure can lead to cognitive deficits, learning challenges, and behavioral disorders, including symptoms commonly associated with attention deficit hyperactivity disorder (ADHD) and other conduct-related conditions. For adults, chronic exposure to lead has been linked to memory impairments, diminished cognitive abilities, and a heightened likelihood of developing neurodegenerative disorders such as Alzheimer’s disease [45]. Lead has been shown to induce vascular damage and promote the development of atherosclerosis, a pathological condition characterized by arterial hardening, thereby elevating the risk of cardiovascular events such as myocardial infarction and cerebrovascular accidents [46].

Cadmium is classified as a human carcinogen and is harmful to various organs, particularly the kidneys, bones, and lungs [47]. Cadmium exposure can occur through various environmental and occupational routes, with sources including contaminated food, water, air, and tobacco smoke, as well as certain industrial activities [29,45]. Cadmium is absorbed by plants, mainly through contaminated soil, and accumulates in certain crops such as rice, leafy vegetables, and root vegetables, as well as seafood, especially shellfish and certain fish like tuna, due to contamination in aquatic environments [48].

Tobacco smoke is another significant source of cadmium exposure. Cadmium is present in tobacco plants because the metal accumulates in the plants’ soil [28]. Smoking results in direct inhalation of cadmium, which can lead to significant exposure, particularly in long-term smokers [33]. It is estimated that smokers have a higher body burden of cadmium compared to non-smokers [49,50]. Chronic exposure to cadmium primarily affects the kidneys, leading to kidney dysfunction and, in severe cases, kidney failure [47]. Prolonged exposure to cadmium can weaken bones, increasing the risk of fractures. This condition is known as Itai-Itai disease, which was first reported in Japan and is characterized by severe pain, bone fractures, and kidney dysfunction due to chronic cadmium exposure [46,51]. Chronic exposure to cadmium has been associated with increased blood pressure, which may contribute to the development of cardiovascular disease over time [52]. Cadmium exposure has also been shown to affect reproductive health, particularly in men, where it can lead to reduced sperm quality and fertility, and may also affect fetal development in pregnant women [53,54,55]. Table 3 summarizes the routes from absorption to excretion.

Risk is not uniformly distributed. Vulnerability is shaped by developmental stage (fetuses and children are most susceptible to neurotoxicity), physiologic state (pregnancy mobilizes maternal bone lead; CKD impairs excretion of Cd and Hg) [56], nutrition (iron deficiency increases Pb absorption; low calcium and zinc may exacerbate retention) [57], and co-exposures (tobacco smoke as a cadmium source, alcohol and medications affecting hepatic or renal handling) [58]. Social determinants—housing quality, access to safe water, food security, and occupational protections—modulate the likelihood of exposure and the capacity to mitigate it. Clinicians should couple medical history with a targeted exposure inventory that asks about housing age and renovations, water source and filtration, fish species and frequency, rice origin and preparation, workplace controls and PPE, recreational shooting, hand-to-mouth behaviors in children, cosmetics and folk remedies, and household contacts who may carry contaminants home from work.

Regulatory thresholds and health-based guidance vary by jurisdiction and are updated periodically. Importantly, no blood lead threshold has been identified as ‘safe’ for children, and both biomarker magnitude and clinical context should guide clinical action [59]. MeHg advisories are typically intake-based, translating to hair or blood benchmarks in surveillance [60].

Cadmium risk is often assessed using urine cadmium (normalized to creatinine) as a marker of body burden and as a tubular effect indicator (e.g., β2-microglobulin) [61].

Inorganic arsenic is best evaluated with urine speciation (iAs, MMA, DMA) to distinguish toxic species from non-toxic seafood arsenicals [62]. Equally crucial is risk communication: providing precise, culturally competent advice on safer fish choices, rice preparation to reduce arsenic, home lead hazard control (wet methods, HEPA vacuuming, interim controls), workplace hygiene (on-site laundering, changing areas), and smoking cessation. The objective is to reduce ongoing exposure before or alongside any medical intervention.

However, when humans experience high levels of metal in their bodies, chelation therapy is recommended. Chelation is only recommended when toxicity symptoms are present, and lab tests confirm toxic levels. There are specific concentrations of heavy metals for recommended chelation therapy (see Table 4) [63,64].

Instead, treatment decisions should integrate the metal species, biomarker level, symptoms, exposure context, and the likelihood that ongoing exposure can be stopped. For children with lead exposure, major clinical guidance supports chelation at blood lead concentrations of 45 µg/dL or higher, while recognizing that harm occurs well below this threshold. For mercury and arsenic, chelation may be appropriate in selected cases of acute or symptomatic poisoning, depending on speciation and timing. For cadmium, routine chelation is generally not recommended, because evidence of benefit is limited and some chelators may increase toxicity. Overall, risk assessment for heavy metal exposure should be species-aware, exposure-specific, and clinically contextualized. The most useful framework links source identification, biomarker interpretation, patient vulnerability, and practical exposure reduction strategies before or alongside any consideration of chelation.

## 4. Chelation Therapy–State of the Art

Chelation therapy, derived from the Greek word *chele* (“claw”), is a medical treatment used to remove heavy metals or other toxic substances from the body by binding them to chelating agents [65]. This treatment is primarily used to treat poisoning from metals such as mercury, lead, cadmium, and arsenic and to manage metal overload disorders, such as excess copper or iron. Chelation therapy works through the chemical properties of chelating agents, which contain specific functional groups, such as amines, thiols, or carboxyl groups, that can bind to metal ions. These interactions form stable, water-soluble complexes that neutralize the metal’s toxic effects. The process consists of three key stages: chelating agents bind to metal ions in the bloodstream or tissues, a stable complex forms between the agent and the metal, and the resulting complex is eliminated from the body through urine or feces. This process effectively decreases the toxic metal load and alleviates its harmful impact on the body.

Several endogenous and exogenous sulfur-containing compounds have metal-binding or redox-active properties, including alpha-lipoic acid (ALA), cysteine (Cys), and glutathione (GSH) (see Table 5). These are naturally occurring compounds in the human body, but they are not established primary chelators in routine clinical toxicology [67,68,69,70]. ALA is widely used as a nutritional supplements, especially in the US, in tablet, capsule, and liquid forms, and have been used as antioxidants to improve cellular glucose utilization in metabolic disorders and type-2 diabetes [69]. In 1966, Germany approved ALA as a drug for the treatment of diabetic neuropathy, and it is available as a non-prescription pharmaceutical [71,72,73]. Among the clinically established chelating agents are dimercaprol (BAL), dimercaptosuccinic acid (DMSA; succimer), calcium disodium EDTA, and dimercaptopropanesulfonic acid (DMPS), although approval status varies by country. In the United States, succimer, calcium disodium EDTA, and dimercaprol are FDA-approved for specific indications, whereas DMPS is not FDA-approved [74,75]. Chelation therapy is best understood as a targeted tool within a broader exposure-reduction strategy, not a panacea. The decision to chelate should follow a structured appraisal: *(1) Which metal and species are implicated? (2) What is the clinical scenario—acute, subacute, or chronic? (3) Are there symptoms or objective organ injury? (4) What are the biomarker levels and their trajectory? (5) Are exposures controlled? (6) Do the anticipated benefits outweigh the risks for this patient in this setting?* Across Hg, Pb, Cd, and As, indications, drug selection, dosing, and monitoring differ in clinically meaningful ways.

Alpha-lipoic acid (ALA) and its reduced form dihydrolipoic acid (DHLA) are sulfur-containing compounds with antioxidant and potential metal-binding properties. Their chemical behavior is influenced by carboxyl and thiol groups, and DHLA is generally the more reactive metal-binding form. Although ALA has been discussed as a possible adjunct in mercury, cadmium, and other metal toxicities, its clinical role remains limited by poor water solubility, variable bioavailability, and the fact that much of the evidence is mechanistic or experimental rather than based on established therapeutic use.

By contrast, DMSA and DMPS are the principal clinically relevant thiol chelators [76,77,78]. Both contain reactive sulfhydryl groups that form stable complexes with toxic metals, but they differ in solubility, elimination, and typical use: DMSA is widely used orally, especially for lead and selected mercury or arsenic exposures, whereas DMPS is more water-soluble and is used particularly for inorganic mercury and arsenic in settings where it is available [79,80]. In the United States, however, DMPS is not FDA-approved, and its use is more limited than that of succimer, dimercaprol, or calcium disodium EDTA.

Endogenous thiols such as L-cysteine and glutathione also contain sulfhydryl groups and play major roles in redox regulation, antioxidant defense, and cellular protection. However, their direct chelating effects are limited, so they should be regarded mainly as supportive biochemical defenses rather than primary therapeutic chelators [81,82]. Overall, the section supports a practical distinction between experimentally interesting sulfur-containing compounds, physiologic thiols with mainly protective roles, and evidence-based chelators that have clearer clinical applications [63].

**Table 5:** ALA, DHLA, DMSA, DMPS, Cys and GSH—selected chemical features relevant to metal binding.

Compound	Type	Key Groups	Representative pKa	Solubility/Route	Main Metals Chelated	Key Role/Practical Use	Key Cautions/Limitations	Refs.
**ALA**	Adjunctive redox-active compound	Disulfide + carboxylate	~4.7–5.1	Low solubility	Hg, Cd, Pb, As (possible)	Antioxidant; possible adjunct chelator	Poor bioavailability; GI/skin effects	[63,74]
**DHLA**	Reduced ALA form	2 thiols + carboxylate	~4.8/~10.7	More hydrophilic	Hg, As, Cd, Pb	Reactive metal-binding form	Limited clinical use data	[63,75]
**DMSA**	Clinical chelator	2 thiols + 2 carboxylates	~3.7/4.9; ~9.5–10.5	Moderate; PO	Pb, inorganic Hg, As	Oral chelator; common for Pb	GI upset, rash, rare cytopenias	[68,83,84]
**DMPS**	Clinical chelator	2 thiols + sulfonate	~8.5–9.5	High; PO/IV	Inorganic Hg, As, Pb	Useful for Hg/As	Hypersensitivity; renal monitoring	[68,70,85]
**CaNa_2_EDTA**	Clinical chelator	Polycarboxylate	-	IV (±IM)	Pb	Severe Pb toxicity	Nephrotoxicity; correct salt required	[79,86]
**BAL**	Legacy chelator	Dithiol	-	IM	Severe Pb, acute inorganic As	Bridge in severe poisoning	Painful; HTN; G6PD caution	[80]
**L-Cys**	Physiologic thiol	Thiol + amine + carboxylate	~1.7/~10.8/~8.3	Endogenous	Weak metal binding	Redox support; H2S precursor	Adjunctive only	[64,87]
**GSH**	Physiologic thiol	Peptidic thiol + carboxylates	~2.1/~9.6/~8.7	Hydrophilic endogenous tripeptide	Limited direct chelation	Antioxidant defense	Not a primary chelator	[67,81,82]
**Physiologic thiols**	Supportive agents	Sulfhydryl groups	-	PO/IV (varies)	Limited chelation	Redox support	Do not replace chelation	[88,89,90]

Chelation efficacy reflects thermodynamics (stability constants), kinetics (on- and off-rates), ligand availability in target tissues, and continuous removal of the formed complex [91,92]. At equilibrium for a 1:1 complex M + L ⇌ ML, the stoichiometric stability constant is *K*_1_ = [ML]/([M][L]) [93]. In vivo, conditional stability (Keff) depends on pH, competing ligands, and matrix effects; the chelatable fraction scales with Keff and the effective tissue concentration of L. Practically, therapy aims to keep complexation and excretion moving forward particularly through urinary clearance, rather than to reach equilibrium [94]. If a chelating agent contains several coordinating groups, the equilibrium behavior becomes more complex. In biological systems, the total concentrations of free L and M are often much lower than the total [L_t] and [M_t], and this is better described by the conditional or effective stability constant, K_eff. In theory, the chelatable metal fraction, [ML], is determined by K_eff and the tissue concentration [L_t] of the chelator. Efficient chelation therefore depends not only on affinity, but also on tolerability, tissue access, and continued elimination of the metal–ligand complex [95].

Regarding the chemical stability of a therapeutic chelate, it is worth noting that the initial assessment of a ligand’s (L) affinity for a toxic metal can be inferred using the Hard-Soft Acid-Base (HSAB) (see Table 6) [96]. Lewis defined an acid as an electron-pair acceptor and a base as an electron-pair donor. Based on this definition, positively charged metal ions are classified as acids because they function as electron acceptors. Forming a solvated (hydrated) metal ion creates a complex where water molecules act as electron donors, making H_2_O a Lewis base. Similarly, many oxygen-containing compounds also serve as Lewis bases.

In aqueous solutions, metal ions exist in solvated forms, and during chelation or complexation, water molecules in the solvation shell are replaced by another ligand (another Lewis base), forming a metal complex [96]. Lewis’s acids and bases can be categorized as “hard” or “soft.” Hard metal ions are generally small, highly charged, and less polarizable, whereas soft metal ions, such as Cu^+^, Hg^2+^, and Pt^2+^, are relatively larger and do not hold their valence electrons as tightly.

The stability of metal complexes and chelates depends on the hardness or softness of the metal ion and the ligand, as explained by Pearson in his Hard-Soft Acid-Base (HSAB) theory. Stable complexes generally result from interactions between hard and hard bases or soft acids and soft bases. For instance, oxygen-donor ligands form stable complexes with harder acids such as Fe(II) and Fe(III).

In contrast, soft ligands, such as those containing sulfur or selenium, form stable complexes with soft metals like mercury, cadmium, copper(I) and mercury species. Hard-hard complexes are predominantly electrostatic, as exemplified by the iron-oxygen bond, whereas soft-soft complexes exhibit more covalent bonding, such as the mercury-thiol bond.

Metals such as mercury and arsenic have a notably high electronegativity (approximately 2.0 on the Pauling scale), predisposing them to covalent bonding with carbon and sulfur. This property accounts for the abundance of organomercurial and organoarsenic compounds [97].

## 5. Toxicokinetics and Toxicodynamics

The ADME processes-absorption, distribution, metabolism, and excretion—vary significantly among Hg, Pb, Cd, and As, influencing biomarker choices and treatment strategies [98] (see Table 7). Elemental mercury vapor (Hg^0^) is rapidly absorbed through the lungs and converted in tissues to inorganic mercury, which accumulates in the kidneys and CNS. In contrast, ingested inorganic salts (Hg^2+^) are poorly absorbed in the gastrointestinal tract [99]. Methylmercury, bound to cysteine, is efficiently absorbed via amino acid transporters in the gut, crosses the blood–brain and placental barriers, undergoes enterohepatic recirculation, and builds up in neural tissues [100].

Lead absorption is greater in children compared to adults and rises with iron or calcium deficiency [101]. After initial distribution to soft tissues, most of the body’s lead burden is stored in bone, with half-lives spanning years to decades and the potential for physiological remobilization during pregnancy, lactation, osteoporosis, and hyperthyroidism [102].

Cadmium’s gastrointestinal (GI) absorption is generally modest but increases during deficiency states and through shared metal transporters [103]. Its complexation with metallothionein promotes accumulation in the renal cortex and results in prolonged half-lives, explaining why urinary cadmium remains detectable long after exposure has decreased [52].

Inorganic arsenic is absorbed efficiently, then methylated in the liver to MMA and DMA, and mainly excreted via urine [104]. Variations among individuals in methylation capacity, such as higher levels of MMA, are linked to increased susceptibility to arsenic toxicity [105].

Toxicodynamics involve common pathways such as thiol binding, oxidative stress, and mitochondrial dysfunction, but they also include specific targets for each agent [106]. Lead disrupts voltage-gated calcium channels and heme synthesis by inhibiting δ-aminolevulinic acid dehydratase, leading to neurocognitive issues and anemia [107].

Cadmium affects redox balance, damages proximal tubular function, and promotes osteotoxicity by disrupting calcium and vitamin D metabolism [108]. Mercury harms neurons and podocytes through oxidative and immune pathways, with methylmercury (MeHg) being especially neurotropic [91].

Arsenic, particularly in its trivalent form, binds to enzyme complexes dependent on lipoic acid and interferes with signal transduction, angiogenesis, and DNA repair, explaining its vascular and carcinogenic effects [92]. Understanding these mechanisms helps in interpreting biomarkers and developing strategies to support nutrient status and reduce iatrogenic risks during treatment [109].

**Table 7:** ADME snapshots that inform testing and therapy.

Element	Key Absorbing Route(s)	Distribution Highlights	Biotransformation	Biological Half-Life	Preferred Clinical Biomarkers	Refs.
**Hg**	Hg^0^: inhalation; MeHg: GI; Hg^2+^: limited GI	Kidney, CNS; MeHg → neural tissue	Hg^0^ → Hg^2+^ (oxidation); MeHg enterohepatic	Inorganic Hg: ~1–2 months; MeHg: ~50 days	Blood Hg (recent MeHg/Hg^0^); Urine Hg (inorganic); Hair (MeHg, months)	[98]
**Pb**	GI (↑ children, Fe/Ca deficiency); inhalation	Blood → soft tissues → bone (primary reservoir)	No meaningful metabolic transformation	Blood: often approximated as ~1 month; elimination is multiphasic	Venous BLL; (bone Pb K-XRF in research)	[101]
**Cd**	GI (↑ with deficiency), inhalation (work)	Renal cortex, liver; metallothionein binding	Limited metabolism; very slow turnover	Kidney/body burden: ~20–30 years	Urine Cd (Cr-corrected); LMW proteins for effect	[108]
**As (iAs)**	GI; inhalation (industry)	Systemic; methylation-capacity dependent	Hepatic methylation → MMA/DMA	~2–4 days (often cited ~4 days)	Urine As speciation (iAs/MMA/DMA); avoid seafood pre-test	[92]

**Abbreviations:** BLL, blood lead level; K-XRF, K-shell X-ray fluorescence; Cr-corrected, creatinine-corrected; LMW, low-molecular-weight; **→**, conversion or transfer to; **↑**, increased absorption/uptake or risk.

## 6. Clinical Manifestations

Clinical signs depend on the dose, exposure duration, species involved, as well as the patient’s age and health conditions [110]. Neurological symptoms can range from slight decreases in executive functioning and attention in children with low-level lead exposure [59] to more serious effects like peripheral neuropathy, tremors, and neuropsychiatric issues associated with mercury [111]. In utero methylmercury exposure is linked to delayed neurodevelopment, which can take years to detect [112]. Arsenic exposure presents with skin changes such as palmar-plantar hyperkeratosis and raindrop hyperpigmentation, alongside vascular problems, peripheral neuropathy, and higher risks of skin, bladder, and lung cancers in people exposed over time [113]. Cadmium primarily causes kidney damage, particularly in the proximal tubules, leading to proteinuria and progressive chronic kidney disease. It also affects bones, causing osteopenia and fractures in severe cases [114].

Acute cases require quick stabilization and focused history-taking (see Table 8). In cases of inorganic arsenic ingestion, symptoms often include severe gastrointestinal issues, dehydration, low blood pressure, QT interval prolongation, and nerve damage [115]. Lead encephalopathy manifests with vomiting, ataxia, seizures, and mental changes; these require urgent chelation therapy and source removal [116]. Inhalation of elemental mercury vapor can cause pneumonitis and later neurological problems; early supportive treatment and removing the patient from exposure are essential [117]. Subacute and chronic cases are more variable: symptoms like fatigue, headache, cognitive difficulties, tingling, mild anemia, hypertension, reduced eGFR, and hormonal changes [110]. A detailed exposure history, symptom review related to specific systems, targeted physical exam (checking skin, nervous system, and the characteristic ‘blue-black’ Burton line on gums in lead poisoning), and basic laboratory tests are vital for guiding further testing [118].

Recent real-world case reports and outbreaks further illustrate how heavy metal intoxication continues to present through household, occupational, environmental, medicinal, and behavioral exposure pathways, often with nonspecific early manifestations that delay diagnosis (see Table 9).

Differential diagnosis covers a wide range (see Table 10). When evaluating neuropathy and cognitive issues, consider conditions like diabetes, thyroid problems, B12 deficiency, medication side effects, sleep disorders, and depression [132]. For renal issues, review NSAID usage, contrast exposure, autoimmune diseases, and obstructive uropathy [133]. To distinguish anemia causes, differentiate between iron deficiency, chronic disease, hemolysis, and sideroblastic conditions [134]. Indicators of metal exposure include relevant exposure history, combined neurological and kidney symptoms, skin signs such as in arsenic poisoning, and biomarkers that help identify specific metals [110].

## 7. Clinically Relevant Toxic Dose Ranges and Thresholds

Clinically relevant toxic thresholds help translate exposure and biomonitoring results into decisions, but they should be interpreted as action-oriented reference values rather than absolute cutoffs (see Table 11). Toxicity varies according to chemical species, route, duration, age, nutritional status, pregnancy, comorbidity, and symptom burden. For mercury and arsenic, speciation is especially important because organic and inorganic forms differ substantially in toxicokinetics and clinical implications. For lead, no safe pediatric blood concentration has been established, so even relatively low values may justify exposure investigation and follow-up. For cadmium, chronic renal risk may occur at lower urinary levels than older regulatory models suggested. Table 11 summarizes clinically relevant acute and chronic thresholds for Hg, Pb, Cd, and As together with the preferred matrix and major interpretive caveats.

## 8. Diagnostics and Monitoring

The diagnostic approach starts with an exposure history and moves to specific biomarkers selected to address a particular clinical question such as recent exposure, body burden, or effect [138] (see Table 12). For lead, venous blood lead level (BLL) remains the main test for recent exposure and risk assessment, and capillary results must be confirmed by venous testing to prevent false positives caused by surface contamination [139]. For mercury, whole-blood mercury indicates recent methylmercury (MeHg) and elemental exposure, while urine mercury provides a better measure of inorganic forms and renal burden [140]. For cadmium, urine cadmium (adjusted for creatinine) acts as a marker of body burden, supplemented by low-molecular-weight protein markers (e.g., β2-microglobulin) to assess tubular effect [141]. For arsenic, total urine arsenic is not meaningful without speciation, laboratories should report inorganic arsenic (iAs), monomethylarsonic acid (MMA), and dimethylarsinic acid (DMA) separately and advise patients to avoid seafood for at least 48 h before testing to reduce arsenobetaine interference [142,143].

Speciation becomes clinically significant when it influences management decisions. Differentiating between MeHg and inorganic Hg helps determine whether dietary advice or workplace safety measures are more urgent [140]. A high MMA-to-DMA ratio for arsenic indicates reduced methylation ability and a potentially increased risk [144,145]. Bone lead levels measured by K-XRF serve as a research tool indicating long-term exposure, which can aid in epidemiological studies, though they are rarely decisive in individual clinical assessments [146]. Hair analysis for MeHg is suitable when sampling and cleaning protocols are strict; however, results should be interpreted considering local fish consumption habits and cosmetic treatments [140].

Different biological matrices answer different clinical questions, and no single specimen is universally superior for all metals or exposure scenarios. Blood is generally most useful for recent or ongoing exposure, particularly for lead and some forms of mercury. Urine is often preferred for inorganic mercury, cadmium body burden, and arsenic speciation, especially when renal handling and recent exposure are relevant. Hair can provide a longer-term exposure signal, especially for methylmercury, but is more vulnerable to external contamination and requires careful sampling and interpretation. Sweat has limited validated clinical utility and should not be used as a primary diagnostic matrix for toxic metal exposure. A comparative understanding of matrix strengths and limitations can improve test selection, reduce misinterpretation, and better align laboratory findings with clinical decisions.

**Table 12:** Clinical Utility of Biomonitoring Matrices for Heavy Metal Exposure Assessment.

Matrix	Best Use	Strengths	Limitations	Practical Applications	Refs.
**Blood**	Recent or ongoing exposure; first-line testing for Pb and useful for Hg	Standardized; clinically familiar; reflects recent exposure	Less informative for long-term body burden for some metals	Venous BLL; whole-blood Hg for recent MeHg/Hg^0^ exposure	[142]
**Urine**	Body burden or excretion patterns; preferred for Cd, inorganic Hg, and As speciation	Useful for renal handling; supports speciation	Affected by hydration; may require creatinine correction	Urine Cd; urine Hg for inorganic exposure; urine iAs/MMA/DMA	[61]
**Hair**	Longer-term exposure pattern, especially MeHg	Reflects exposure over weeks to months; noninvasive	External contamination; cosmetic treatment effects	Long-term methylmercury exposure assessment	[143]
**Sweat**	Limited or investigational use only	Noninvasive in theory	Poor standardization; limited clinical validation	Not recommended as a primary routine diagnostic matrix	[147]

Pitfalls include relying on unvalidated ‘provoked’ urine tests after chelator challenge, which can nonspecifically increase urinary metal excretion and lead to potential misclassification of exposure [148] (see Table 13). Urine test results should be adjusted for creatinine levels, and clinicians must consider hydration status [138]. Pre-analytical factors such as tube type, contamination prevention, and chain-of-custody procedures in cases with occupational or legal consequences are vital to prevent misinterpretation [149,150]. Ultimately, clinicians should interpret biomarker results alongside decision thresholds that reflect symptoms, ongoing exposure, and patient preferences [151]. Testing without a clear management plan may cause unnecessary anxiety and treatment [152].

## 9. Supportive and Preventive Management of Toxic Metal Exposure: Source Control, Nutrition, and Rational Use of Chelation

**Figure 2:**
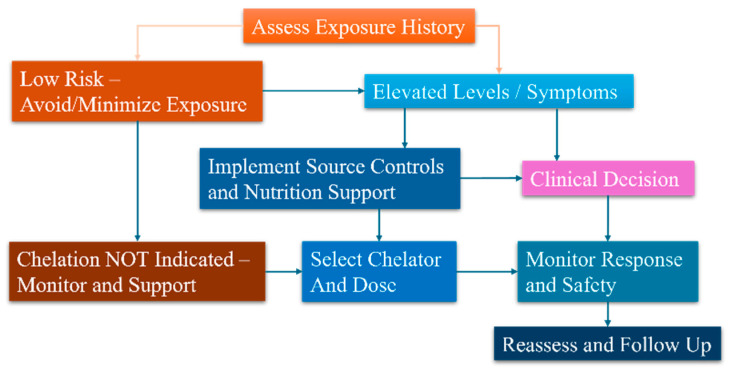
Clinical decision pathway for evaluating toxic exposure, beginning with exposure history and risk assessment, followed by source control, supportive care, chelator selection when indicated, and ongoing monitoring with reassessment.

Managing exposure to mercury (Hg), lead (Pb), cadmium (Cd), and arsenic (As) starts with understanding that removing the source and providing careful supportive care often leads to better outcomes, and poses fewer risks than pharmacological treatments [110,155] (see Figure 2). The key steps include: (1) confirming exposure and its circumstances, (2) reducing or stopping ongoing exposure, (3) addressing host factors such as nutrition, other health conditions, and medications, (4) promptly treating acute complications, and (5) consulting chelation therapy only if evidence indicates that benefits outweigh potential harms. Since these toxins affect multiple organs and often occur together, clinicians should plan a coordinated approach involving individual, household, workplace, and public health measures.

Source control is the anchor. For the home, practical steps include identifying and stabilizing deteriorating lead-based paint (use of wet methods rather than dry sanding; high-efficiency particulate air [HEPA] vacuuming, interim controls, professional abatement when indicated), flushing stagnant plumbing and replacing lead service lines or leaded brass fixtures where feasible, using certified point-of-use filters for drinking and cooking water, and addressing imported ceramics, spices, cosmetics, or traditional remedies implicated in outbreaks [156]. For diet, pair exposure reduction with nutrition: correct iron deficiency (reduces gastrointestinal absorption of lead), ensure adequate calcium and zinc intake, guide safer fish choices and portion sizes to minimize methylmercury while maintaining omega-3 benefits, and discuss rice sourcing and preparation techniques (e.g., rinse/soak, excess-water cooking) for arsenic [157,158,159,160]. For tobacco exposure, smoking cessation is a cadmium reduction strategy with significant co-benefits [161]. At work, hierarchy-of-controls thinking applies to eliminate or substitute metals where possible, implement engineering controls (local exhaust, process enclosure, wet methods), administrative controls (rotation, hygiene policies), and personal protective equipment (fit-tested respirators, gloves), provide on-site showers and changing areas to prevent ‘take-home’ contamination [110].

Acute management begins with ABCs (airway, breathing, circulation), decontamination when necessary, and early contact with a regional poison center or toxicologist [117]. For acute inhalational exposure to Hg vapor, remove the patient from the source, administer supplemental oxygen, consider corticosteroids for chemical pneumonitis on a case-by-case basis, and observe for delayed neuropsychiatric effects [162]. In cases of acute inorganic arsenic ingestion, provide aggressive fluid resuscitation, antiemetics, electrolyte repletion (notably potassium and magnesium in diarrhea or QT interval prolongation), cardiac monitoring, and consider chelation early [163]. Acute lead encephalopathy is a neurological emergency requiring management of intracranial pressure, seizure control, and prompt initiation of guideline-based chelation combined with environmental assessment [164]. For suspected mixed metal exposures, such as e-waste or battery recycling, expand the diagnostic scope and avoid focusing solely on one analyte [75].

Supportive and preventive measures include targeted laboratory and clinical monitoring [110]. Establish baseline levels for complete blood count, electrolytes, renal and hepatic function, and urinalysis; consider adding β2-microglobulin or retinol-binding protein if cadmium nephrotoxicity is suspected [108]. Schedule repeat testing based on the expected kinetic timelines: weeks to months for mercury and lead, months to years for cadmium, and days to weeks for arsenic due to its shorter biological half-life but ongoing exposure in endemic areas [110]. Link each lab test to a specific clinical decision, for example, if blood lead remains high despite interventions, escalate home remediation efforts; if urinary inorganic mercury levels decline with mild symptoms, focus on source control rather than chelation [110].

Nutritional and pharmacologic adjuncts require careful framing. In iron-deficient children, iron repletion decreases lead absorption and supports hematologic health [165]. Adequate intake of calcium and zinc can lessen intestinal absorption and prevent displacement from key enzymes. Selenium can influence mercury toxicity on a biochemical level but is not a chelator and should not be relied upon as a standalone detox method [87]. Antioxidants like alpha-lipoic acid (ALA) and glutathione (GSH) are involved in redox homeostasis but lack proven clinical efficacy as primary treatments for heavy-metal poisoning. They may be used as nutritional support in certain cases, but it is important to clarify they do not replace chelation when necessary. Patients should be advised honestly about internet-promoted detox regimens, highlighting the importance of evidence, safety, and reducing exposure [148].

Special populations need customized plans. During pregnancy, focus on prevention and exposure control; avoid chelation unless maternal toxicity is life-threatening and specialist advice is sought [164]. In pediatrics, due to developmental sensitivity and narrow therapeutic margins, accurate dosing and close monitoring are essential when administering any medication, collaborate with public health for home hazard management and caregiver training [153]. In chronic kidney disease, reduced excretory function raises the risk of chelator–metal complex buildup and kidney damage; modify doses accordingly, avoid nephrotoxic drugs, and consult nephrology. For workers, management involves medical removal when required, regular biological monitoring, and collaboration with employers on engineering and administrative controls; record fit testing and training. Engagement of mental health, social work, and community health professionals is crucial for effective advice and action consult them early.

Risk communication serves as a standalone intervention. Effective counseling should be specific such as identifying which fish are safer in your area, how to cook rice properly, and which renovation practices to avoid, while also being culturally acceptable and feasible with the family’s available resources. Using written materials in the patient’s language, including pictures of fish species or water filters, and coordinating with community programs (like lead-safe home initiatives or well testing) can enhance adherence. For households with children, address behaviors like hand-to-mouth activity, frequent wet-mopping of floors and window sills, and washing toys and hands [156]. For shooters, promote range hygiene, such as avoiding food on the line, using dedicated shoes and clothing, and laundering immediately. Ensure clinical messaging aligns with public health agencies to prevent conflicting guidance.

Finally, develop a monitoring and follow-up plan that accounts for biomarker kinetics and the progression of health effects [110] (see Table 14). For example, after removing a high-mercury fish from the diet, reassess hair or blood mercury levels after 3–4 months [166]. Following lead hazard control, monitor venous blood lead levels at intervals suited to the baseline level and age [153]. For cadmium-related tubular proteinuria, check urinary markers twice a year. In endemic regions, combine serial urinary speciation with water remediation verification. Conclude by explaining results clearly and connecting them to future steps. The main goal of supportive and preventive care is to avoid chelation therapy unless necessary, reserving such drugs for patients most likely to benefit [148].

## 10. Comparative Effectiveness and Health Economics

Comparative effectiveness analysis aids clinicians in selecting suitable chelators and recognizing when chelation may not lead to better outcomes [110]. The supporting evidence varies depending on the metal involved and the specific clinical situation. For lead, both randomized and observational studies consistently show that chelators reduce blood lead levels; however, neurodevelopmental improvements in asymptomatic children with moderate elevations have not been seen with succimer, despite significant reductions in blood levels [168] (see Table 15). This disconnects between changes in biomarkers and functional outcomes highlights a key principle: factors like timing, age, ongoing exposure, and initial injury influence whether lowering the biomarker results in tangible clinical benefits [169].

In adults with occupational lead exposure and symptoms, or with very high blood lead levels, chelation combined with removal from exposure can enhance hematologic and neurological health; in these cases, improvements in both biomarkers and clinical conditions typically occur when exposure is effectively controlled [168].

For mercury, the strongest evidence for chelation concerns inorganic forms with renal involvement [170]. DMPS and DMSA increase urinary excretion and can improve symptoms when exposure ceases; evidence for methylmercury is weaker, with benefits most plausible at high levels and with persistent symptoms after dietary cessation [85]. For arsenic, chelation clearly improves outcomes in acute inorganic poisoning, especially when initiated early; beyond the acute setting, benefits are less clear, and prevention dominates [170]. For cadmium, the lack of convincing benefit and renal risks tilt the calculus away from chelation in chronic exposures; supportive renal and bone care with exposure reduction is the standard [110].

Head-to-head comparisons are limited but provide useful insights. DMSA and DMPS both mobilize inorganic mercury and arsenic; DMPS might have practical benefits, such as availability in IV form and strong affinity for mercury, especially in severe cases [168]. Conversely, DMSA’s oral tolerability and pediatric safety data are advantages [83]. For lead, DMSA is generally preferred orally in cases without encephalopathy, while CaNa_2_EDTA (±BAL) is reserved for severe cases [168]. BAL remains a rescue therapy, effective but with limitations due to side effects and logistical challenges [79]. No chelator has proven superior in improving functional outcomes for low-level, chronic exposure without symptoms, compared to thorough source removal and supportive care [169].

Health economics considers these decisions at the system level [171]. The expense of chelators is often overshadowed by the costs of hospital care (for BAL and CaNa_2_EDTA), lab tests, clinic visits, and lost productivity [172]. When chelation does not improve functional outcomes, it is unlikely to be cost-effective compared to aggressive exposure reduction, environmental cleanup, and nutritional strategies [173]. However, in true emergencies, such as lead encephalopathy, acute arsenic poisoning, or severe inorganic mercury exposure with renal failure, chelation prevents morbidity and death and proves highly cost-effective despite short-term costs [170]. This approach supports a tiered strategy: invest in prevention and remediation for the many, use chelation only for the few with clear indications, and provide rapid access for the rare, life-threatening cases [174].

Access and implementation are crucial [175]. In low- and middle-income countries and underserved communities, challenges include shortages of chelators like BAL and CaNa_2_EDTA, cold-chain or compounding needs, limited laboratory capacity for speciation, and patient-related costs [175]. Policies that subsidize certified water filters, lead service line replacements, and safer renovation practices often yield higher population health benefits per dollar compared to expanding chelation options [176]. When chelation is necessary, using standardized order sets, dosing calculators, and checklists helps minimize errors and waste [177]. Training clinicians to identify red flags such as encephalopathy, GI shock in arsenic poisoning, or renal injury from inorganic mercury can shorten the time to treatment and lead to better health outcomes [178].

Stewardship combines clinical ethics and economics [179]. An effective program rests on three pillars: (1) diagnostic accuracy, confirming the species, verifying ongoing exposure, and avoiding provocation tests; (2) treatment discipline, using clear indications, weight-based dosing, lab monitoring, and stopping rules; and (3) prevention as a priority [148], documenting source control, notifying public health, and protecting workers. Quality metrics may include the time from diagnosis to exposure mitigation, the percentage of chelation episodes meeting strict criteria, adverse drug event rates, and patient-reported outcomes like symptom scores and function at follow-up [180]. Integrating these metrics into electronic health records and public-health reports supports accountability and program evaluation [181].

Decision-support tools can convert comparative evidence into practical bedside decisions [182]. Simple algorithms, based on factors like metal levels, biomarker ranges, symptom clusters, or pregnancy/CKD status, help determine whether to (a) increase exposure control and monitoring, (b) start outpatient oral chelation with close follow-up, or (c) escalate to inpatient parenteral therapy [183]. Cost-aware versions of these algorithms can factor in local drug availability, lab access, and social support systems [184]. When clinicians and public health partners use consistent pathways and terminology, the healthcare system can efficiently direct patients to the appropriate intervention at the optimal time, thereby maximizing health benefits relative to costs [185].

**Table 15:** Scenario-based clinical decision framework for efficient management of toxic metal exposure.

Scenario	Best Option(s)	Why This Choice Is Efficient	Refs.
**Child with moderate BLL, asymptomatic, ongoing hazards**	Aggressive source control ± DMSA only if indicated	Neuro outcomes hinge on exposure removal, not the biomarker alone	[186]
**Adult worker with high BLL and neuropathy**	Chelation + medical removal + workplace controls	Biomarker and clinical gains align when exposure stops	[136]
**Acute iAs ingestion with shock**	Immediate BAL → DMPS/DMSA + ICU care	Early action prevents morbidity/mortality	[187]
**Chronic Cd nephropathy**	No chelation; renal/bone support + exposure reduction	Chelation adds risk without proven benefit	[188]
**Inorganic Hg with renal injury**	DMPS/DMSA after source control	Symptom improvement plausible; monitor kidneys closely	[119]

## 11. Emerging and Adjacent Therapies

Emerging and related therapies aim to address two major limitations of current approaches: (1) the limited selectivity of chelators that can remove essential metals and lead to side effects, and (2) the weak connection between molecular diversity (such as speciation, host genetics, and microbiome) and personalized treatment [189]. The research field is vibrant across materials science, pharmacology, and systems toxicology, with several themes expected to shape clinical practices in the coming decade [190].

Next-generation chelators and delivery systems emphasize specificity, tissue targeting, and safety [190]. Polymeric and nanoparticle scaffolds can display multiple thiol, dithiol, or hydroxamate groups at precise intervals, increasing affinity for target metals while reducing unintended binding to zinc and copper [191]. Ligands can be adjusted to prefer certain oxidation states (such as Hg^2+^ versus organomercury) or to operate effectively across physiological pH ranges [192]. Encapsulation methods, including liposomes, polymeric micelles, or dendrimers, can direct chelators to renal or hepatic tissues, lowering systemic exposure and potentially enhancing the therapeutic window [84]. Prodrugs that activate in oxidizing or acidified environments (like inflamed proximal tubules) provide localized activity with reduced systemic toxicity [193]. Although still in early stages, these approaches indicate that future chelation therapy may resemble targeted oncology, precise, compartmentalized, and accompanied by biomarkers [194].

Biological and catalytic methods utilize nature’s innate metal management systems [195]. Engineered metallothioneins, heme-oxygenase mimetics, or peptide sequences with high affinity for Hg, Pb, or Cd can bind metals for removal or aid in transfer to external chelators [195]. Research is also ongoing into catalysts that transform more toxic metal species into forms easier to eliminate, such as organomercury demethylation or arsenic oxidation [196]. Additionally, microbiome-based approaches aim to decrease intestinal absorption or convert metals into less bioavailable complexes [197]. While microbiome modulation through fecal or oral methods is still emerging, it is promising due to the gut’s key role in MeHg uptake and enterohepatic cycling [198].

Precision toxicology combines genomics, epigenomics, and metabolomics with exposure science to assess risk and customize treatment [198]. Variations in metallothionein genes, transporters, or enzymes like AS3MT that are involved in arsenic methylation influence body burdens and health outcomes; similarly, differences in calcium and iron metabolism affect lead kinetics [36]. In clinical practice, this could lead to risk-based intervention thresholds or choosing between DMPS and DMSA, depending on which is predicted to yield a better response or fewer side effects for a patient [36]. Metabolomic profiles may also help distinguish symptomatic from asymptomatic individuals with similar biomarker levels, indicating who might benefit from chelation therapy rather than just intensified prevention [199].

Extracorporeal and device-based therapies are specialized but important options [200]. Intermittent hemodialysis and hemoperfusion play a role in severe inorganic mercury or arsenic poisoning with renal failure, acting as supportive measures until chelation and source control are effective [201]. Future devices might feature metal-selective adsorbents or resin cartridges tailored to specific speciation profiles, enhancing the removal of chelator–metal complexes or free ions during critical short periods [202]. However, practicality remains a challenge: these methods are resource-intensive and compete with more scalable prevention approaches [203].

Adjacency is important: interventions beyond chelators can effectively reduce risk [204]. Adequate selenium levels may help buffer some mercury effects at the enzyme level; the status of iron, calcium, and zinc influences the intestinal absorption of lead and cadmium [205]. Ongoing development in redox modulation drugs shows promise, though clinical outcomes still need validation [195]. In public health, innovations such as point-of-use filtration (like arsenic-selective media and lead-capturing cartridges) and rapid field testing for speciation could revolutionize detection and prevention, lessening reliance on downstream treatments [206]. Digital health tools that monitor fish consumption, rice sources, and water filter upkeep can help turn advice into lasting behavior change across populations [203].

Translation challenges are expected: demonstrating not only biomarker changes but also concrete health outcomes; maintaining manufacturing quality and stability for complex biologics and nanomaterials; aligning pricing and reimbursement with public health advantages; and ensuring equitable access so that high-risk communities receive early, not delayed, access [206]. Despite these hurdles, the outlook is optimistic: more targeted and safer chelation therapies combined with molecular and environmental precision, integrated into a prevention-focused ecosystem [197].

## 12. Guidelines and Practice Pathways

Guidelines and practice pathways convert scattered evidence into clear, actionable steps for clinicians, public health workers, and patients [153]. Since Hg, Pb, Cd, and As vary in forms, behavior, and clinical effects, these pathways must be specific to each metal while maintaining a common framework: screen if risk exists, confirm with the appropriate test, control the source, treat the patient, focusing on individual needs rather than just numbers, and set measurable follow-up goals [207]. A good pathway is concise enough to display on a clinic wall yet detailed enough to address special populations and escalation procedures.

A standardized clinical algorithm starts with assessing exposure risks. In pediatrics, screen for lead exposure by considering housing age, renovation history, and caregiver occupational exposures; perform a venous BLL if there is identified risk or if required by local guidelines [153]. To evaluate methylmercury risk, check fish species and how often they are consumed; for inorganic mercury, inquire about occupational environments such as mining, dentistry, or artisanal gold work, as well as spills and hobbies [208]. For cadmium, ask about tobacco use and occupational history related to batteries or smelting; for arsenic, investigating well water sources, residence in endemic areas, and rice intake [209]. When a risk is plausible, order specific tests such as venous BLL; whole blood or urine mercury depending on suspected species; urine cadmium with creatinine adjustment and markers of tubular damage; and urine arsenic speciation after 48–72 h of avoiding seafood.

Decision nodes should clearly specify action thresholds and priorities. If BLL is significantly elevated or symptoms are evident, start chelation according to protocol (DMSA for moderate cases, BAL + CaNa_2_EDTA for encephalopathy) while accelerating home inspection and remediation. For elevated whole-blood mercury levels alongside a diet high in MeHg, focus on counseling to stop consuming high-mercury fish, consider hair testing to track declines, and reserve chelation for severe or ongoing cases [170]. When urine inorganic mercury is high with signs of kidney involvement, select DMPS or DMSA and monitor renal function. If urine arsenic shows high inorganic fractions and MMA/DMA ratios indicating poor methylation, combine water remediation with chelation for acute toxicity; otherwise, prioritize prevention and cardiovascular risk management [154]. For elevated urine cadmium and tubular proteins, avoid chelation, but increase exposure controls and address CKD risk factors such as blood pressure, blood sugar, and bone health [209].

Practice pathways should include safety checks. Before chelation therapy, obtain baseline CBC, CMP, urinalysis, and pregnancy testing where relevant; screen for G6PD deficiency if BAL is considered; confirm that CaNa_2_EDTA (not disodium EDTA) is ordered to prevent iatrogenic hypocalcemia [210]. Define laboratory monitoring intervals (such as weekly during parenteral treatment, after each oral cycle, and then every 1–3 months) and clinical assessments (for rash, mucositis, transaminitis, neuropathy) [170]. Set stopping criteria: discontinue when biomarkers reach target levels, symptoms resolve, and source control is confirmed; reassess if rebound occurs, with focus on bone lead mobilization and unresolved exposures.

Operationalization is crucial. Develop EHR order sets that automatically select appropriate tubes, tests (including speciation), and timing. Include patient instructions such as “avoid seafood for 72 h before urine arsenic speciation.” Establish referral pathways to public health inspectors, housing agencies, occupational health, and legal aid, especially when landlord or employer cooperation is limited [153]. For pediatric cases, it includes developmental screening and supportive services. For occupational cases, ensure pathways comply with regulatory standards for medical removal, return-to-work criteria, and periodic monitoring [211]. Clearly define roles: primary care handles screening and counseling; toxicology consults oversee chelation decisions; nephrology monitors CKD; obstetrics manages pregnancy care; and public health coordinates environmental interventions [153].

Special populations require dedicated sections in the pathway. Pregnancy: focus on prevention, delay chelation unless maternal health issues justify it, and involve maternal–fetal medicine early [164]. Pediatrics: adopt low thresholds for environmental interventions, ensure careful dosing, and conduct frequent follow-ups [153]. CKD: adjust doses, select agents with safer renal profiles, avoid nephrotoxins, and coordinate care with dialysis if needed [170]. Resource-limited communities: offer practical alternatives like subsidized filters, lists of safer fish relevant to local markets, and affordable rice preparation methods, using community health workers to help overcome language barriers and build trust [212].

Finally, practice pathways should be dynamic documents. Set a regular review schedule (e.g., yearly) to update thresholds, lab techniques, and drug options; monitor metrics such as time to source control, appropriate chelation rate, adverse events, and patient feedback; and include input from patients and frontline clinicians [209]. A successful pathway not only decreases variation but also improves care, accelerates the time to appropriate action, and prevents both undertreatment and overtreatment [169].

## 13. Ethics, Misinformation, and Med-Legal Considerations

Ethics, misinformation, and medico-legal issues are integral to heavy-metal practice, not peripheral [213]. The decisions regarding screening, testing, and chelation occur amidst a landscape filled with internet claims, commercial “detox” products, and legal pressures, which can influence both patients and clinicians in conflicting ways. Ethical practice demands transparency about the evidence limits, a commitment to do no harm, respect for patient autonomy, and fairness for communities disproportionately affected by exposure [214].

The primary ethical issue is to prevent iatrogenic harm caused by unnecessary testing and treatment [215]. Non-validated “provoked” urine tests can show high results that stem from pharmacologic activation rather than true body burden [148]. Using these results to justify chelation can expose patients to drug risks without proven benefit [213]. Ethically, clinicians should order tests that address specific clinical questions and have validated interpretation methods [149]. If patients present results outside these frameworks, clinicians should review them respectfully, explain their limitations, and switch to suitable diagnostic methods [213]. Likewise, chelating asymptomatic patients with low-level biomarkers without ongoing high-risk exposure violates proportionality, as the risks and costs surpass potential benefits [168].

Informed consent for chelation therapy must be comprehensive. Patients should be clear about the expected benefits, such as biomarker reductions and symptom relief in certain cases, as well as uncertainties, like the potential lack of improvement in functional outcomes, especially in cases of chronic low-level exposure [153]. They should also understand available alternatives, including source control, supportive care, and watchful monitoring, along with the associated risks like rash, hypersensitivity, transaminitis, cytopenias, and nephrotoxicity for some agents [214]. The consent process should also address logistical details, such as the need for ongoing lab monitoring, potential mineral supplements, and the meaning of “stopping rules [153].” For pediatric patients, obtaining assent and providing caregiver education are critical, while for pregnant patients, consent must explicitly weigh maternal benefits against fetal risks.

Justice and fairness demand that clinicians identify and address structural obstacles to prevention and treatment. For example, families living in older homes might lack the resources or landlord cooperation needed to fix lead hazards, well owners might not be able to afford arsenic filtration, and workers could face job loss if they report unsafe working conditions. Clinicians are ethically justified to advocate by writing letters supporting remediation, helping patients access public assistance programs, and documenting medical removal needs in occupational cases [153]. On a broader scale, health systems can collaborate with public health agencies to provide bundled services that lower exposure risk, offering these services at no cost to patients who cannot afford them.

Misinformation flourishes amid uncertainty [213]. To combat it, use straightforward language and actively engage with patient concerns instead of dismissing them [216]. Offer clear, practical advice such as which fish to select, how to cook rice, ways to clean window troughs safely, and the appropriate PPE for work, and explain why certain tests or supplements are not advisable [216]. Share trustworthy resources and encourage questions; the aim is to align patients and clinicians around shared priorities, safety, effectiveness, and responsible resource use.

Medico-legal responsibilities include thoroughly documenting exposure history, lab results alongside collection conditions (such as seafood avoidance in arsenic speciation), counseling given, and reasoning for or against chelation [149]. If mandatory reporting is required (like pediatric lead cases), respond promptly and keep records of notifications. In occupational scenarios, record recommendations regarding work restrictions, medical removal, and criteria for return to work; offer clear guidance to employers while safeguarding patient confidentiality [150]. When litigation might be anticipated, consider chain-of-custody protocols and collaborate with the laboratory to ensure proper containers, preservatives, and handling procedures.

Team-based safeguards assist in managing ethical and legal challenges [217]. Create multidisciplinary case reviews for complex or controversial cases, involving toxicology, nephrology, obstetrics, occupational medicine, social work, and legal advice as needed [218]. Use checklists for chelation initiation that cover indication review, baseline laboratory tests, informed consent, public health reporting, and follow-up planning [219]. Incorporate EHR decision support to identify non-validated tests and suggest evidence-based options [220]. Monitor and improve by tracking adverse events, inappropriate chelation occurrences, and patient feedback; share results openly [221].

In summary, a responsible and legally compliant approach to managing heavy metal exposure emphasizes prevention, aligns with evidence, and centers on patient needs [218]. It avoids focusing solely on numerical treatment, prioritizes controlling the exposure source, uses chelation therapy when clinically appropriate, and assists patients in overcoming social and structural barriers that often contribute to initial exposure [221].

## 14. Discussions: Translating Toxicology into Clinical and Public-Health Decision-Making

A key insight from combining Hg, Pb, Cd, and As data across exposure science, clinical toxicology, and public health is that biomarkers are essential but not enough for reliable decision-making [147]. These biomarkers vary based on speciation, matrix, timing, and individual factors; they distill complex and dynamic exposures into a single value that, without proper context, can be misinterpreted (see Table 16). Therefore, the discussion reverts to core principles: (1) understanding the patient’s exposure history and ability to modify it, (2) early recognition of organ-system effects, (3) clear criteria for escalation to treatments like chelation or hospitalization, and (4) ongoing focus on safety and equity [222].

A recurring theme is the nonlinearity between biomarker changes and clinical outcomes. For example, in pediatric cases, succimer reduces blood lead levels, but randomized trials have not shown improvements in neuropsychological endpoints among asymptomatic children with moderate elevations when source control is present [169]. The point is not that biomarkers are useless but that they should be linked with meaningful outcomes, such as development, function, and quality of life, and considered over appropriate biological timeframes [222]. Similarly, with methylmercury, the key “treatment” is dietary counseling and adherence; chelation often adds little, but serial hair or blood mercury tests can confirm reduced exposure and promote behavior changes [167]. Conversely, in cases of acute inorganic arsenic poisoning or severe lead encephalopathy, biomarkers and outcomes align during intensive treatment; here, chelation can be life-saving, and delays can be detrimental [223].

Speciation is central to many clinical decision points [222]. For instance, elevated whole-blood mercury from fish consumption requires a different approach than elevated urine mercury in a dental worker. Similarly, total urine arsenic without speciation is meaningless after a seafood meal [154]. The key takeaway is to order the correct test initially and give pre-analytic instructions, like seafood avoidance, to ensure meaningful results. When labs cannot perform speciation, clinicians should prioritize patient history, conduct repeat tests after avoidance periods, or refer to specialized labs instead of defaulting to empiric chelation [224].

Supportive and preventive management often has more impact than is typically recognized [225]. For various metals, the most effective intervention is to remove or reduce exposure, enabling clinical teams to help the greatest number of patients [147]. Since exposure controls often depend on landlord cooperation, workplace adherence, or infrastructure improvements, clinicians should build partnerships with housing authorities, occupational health services, community organizations, and legal aid [226]. These collaborations facilitate turning recommendations into tangible actions, especially when social factors limit patients’ ability to act independently [226].

The approach to chelation is more complex than simply equating a high number with treatment necessity [223]. Besides emergency situations, a careful trial of exposure control paired with close monitoring often represents the best initial step. When chelation is necessary, success depends heavily on logistics, such as collecting specimens before administering the first dose, choosing regimens suitable for the patient’s age and kidney function, replacing essential minerals, planning laboratory tests, and establishing clear stopping criteria [222]. A structured, checklist-based method helps prevent both unnecessary overuse and unsafe under-monitoring [227]. Although adverse drug events are rare with oral agents, they are more likely with parenteral routes, polypharmacy, CKD, and pregnancy, underscoring the importance of multidisciplinary oversight and explicit consent [228].

Equity runs through the entire concept of clinical effectiveness [225]. The communities most at risk, such as those with older housing, contaminated wells, and unstable jobs, face the biggest hurdles to exposure reduction and follow-up care [226]. In these environments, chelation might be used even when complete remediation is not possible, not because it is the best solution, but because it is the only manageable option [223]. An equitable strategy broadens the range of accessible interventions by incorporating programs that provide remediation, filters, safer renovations, and workplace controls alongside clinical treatments. Tracking and reporting outcomes that are sensitive to equity, such as time to source control, return to work with protections, and access to filters or abatement, helps highlight and address disparities [228].

Another common challenge is the appeal of “detox” stories in media. Patients with vague symptoms and no clear diagnosis may be convinced by marketing that hair analysis or provoked urine tests prove hidden poisoning, and that infusions will cure them. Confronting these beliefs can harm trust. A better approach is to focus on shared goals, such as feeling better and staying safe, explaining what evidence-based tests can and cannot indicate, and propose a plan starting with simple, low-risk actions like keeping an exposure diary, making dietary changes, and implementing safer household practices. If proper testing later detects a specific exposure, chelation options remain available; if not, the patient still gains from safer habits and maintains a strong therapeutic relationship.

Ultimately, the field requires tighter feedback loops linking research and practice [229]. Population-based evidence highlights risks at biomarker levels common in everyday life; clinical trials determine where drugs are effective; implementation science explores how to deliver prevention and care within limited settings [228]. When guidelines integrate all three elements and electronic health records (EHRs) encode them as defaults, everyday care becomes both more consistent and equitable. The conversation thus returns to its starting point: begin with the exposure story, select the appropriate test, implement the most impactful controls, use chelation when beneficial, and focus on measuring what truly matters.

## 15. Conclusions

This review presents a straightforward yet disciplined approach to managing Hg, Pb, Cd, and As exposure, aligning complex toxicology with practical medical application. First, emphasize prevention and source control, as reducing or eliminating exposure offers the greatest benefit with the least risk and cost across various settings. Second, tailor diagnostics to inform decisions by selecting tests that genuinely influence management, such as speciation when it impacts counseling or medication choices; venous confirmation for positive screenings; creatinine-corrected urine for assessing renal burden; and hair analysis only when protocols ensure accurate interpretation. Third, focus on treating patients rather than just numbers. Understand that biomarker reduction alone is not a patient-centered goal, and that functional recovery depends on factors like timing, age, organ involvement, and the effectiveness of exposure control.

Fourth, reserve chelation for patients who are most likely to benefit. Emergency cases, such as lead encephalopathy, acute inorganic arsenic poisoning, or severe inorganic mercury poisoning with renal injury—require prompt, protocolized chelation combined with ICU-level supportive care. In other situations, oral chelation (like DMSA or DMPS) may be appropriate when symptoms and biomarker levels justify the risks and logistical considerations, especially after exposure is controlled. When using chelation, safety is critical: perform baseline labs, follow weight-based dosing, monitor minerals, watch for hypersensitivity, and have clear stopping rules. Fifth, incorporate equity and ethics into care. Collaborate with public health, housing, and employers to align treatment with patients’ real-life circumstances; avoid using unvalidated tests and low-value treatments; and ensure thorough informed consent that respects patient autonomy without shifting undue risk onto those least able to handle it.

For clinicians, three practical algorithms can be derived from these principles. For lead, risk screening should be followed by measurement of venous blood lead level (BLL), implementation of exposure control, and chelation only when symptoms are present or the BLL exceeds age-specific thresholds. Remediation should be confirmed, and patients should be monitored for rebound. For mercury and arsenic, the first step is to identify the chemical species involved. In cases of dietary methylmercury (MeHg) exposure, counseling and exposure reduction are usually the primary interventions. In cases of inorganic mercury or inorganic arsenic (iAs) exposure with organ injury or markedly elevated levels, DMPS or DMSA may be considered, with BAL reserved for severe arsenic toxicity, while renal and hepatic function are monitored throughout treatment. Therapy should be discontinued once biomarker levels decline and symptoms resolve. For cadmium, renal tubular injury should be confirmed using appropriate biomarkers; chelation is generally not recommended, and management should focus on exposure reduction and control of CKD risk factors such as hypertension and hyperglycemia.

Health systems and policymakers can put these findings into practice by investing in upstream measures like replacing lead service lines, ensuring arsenic-safe wells, enforcing safer renovation practices, and implementing workplace controls (see Table 17). Additionally, integrating clinical pathways into EHRs with default orders and referral links can streamline care. Educational initiatives should focus on clinicians, covering topics such as speciation interpretation and pitfalls of provocation testing, and on patients, promoting clear, culturally appropriate behavior changes. Key metrics to monitor include the time taken to achieve exposure control, the proportion of appropriate chelation therapy, adverse events, patient-reported outcomes, and equity indicators like access to remediation.

In summary, current approaches to Hg, Pb, Cd, and As prioritize prevention, evidence-based practices, and patient-centered care. Chelation is viewed as a valuable but limited tool, not a universal solution. Emphasis is placed on diagnostics that inform management decisions rather than those that only satisfy curiosity. When clinicians, labs, and public health agencies coordinate using the same guidelines, patients experience dual benefits: reduced exposures and safer, prompter, and effective treatments when necessary.

## 16. Future Directions and Research Gaps

Future work should focus on clinically actionable advances instead of just expanding descriptive data. Important objectives include developing validated rapid speciation techniques that guide treatment, conducting prospective studies to identify significant low-level outcome thresholds, testing safer and more targeted chelators against existing standards, creating precision-toxicology algorithms that incorporate host susceptibility, and carrying out implementation research to assess equity, scalability, and cost-effectiveness in real-world healthcare environments.

(1) Initial diagnostics for speciation are crucial. Affordable and rapid testing for mercury and arsenic, either at the point of care or with quick results from regional labs would help prevent misclassification and enable faster, appropriate interventions like dietary advice, chelation, or remediation. Research should focus on evaluating new analytical technologies, such as portable mass spectrometry and electrochemical sensors, and determine clinically validated cut-offs linked to health outcomes, not just exposure levels.
(2) Validated clinical endpoints are necessary for low-level exposure. Although many associations at low biomarker levels are statistically significant, they tend to be small, making it difficult to translate these findings into clear clinical thresholds. Prospective cohort studies that include standardized neurocognitive, renal, cardiovascular, and reproductive endpoints along with repeated biomarker measurements and exposure diaries can help clarify dose–response relationships and guide patient-centered decision-making. Additionally, research should explore whether intensive prevention strategies, such as filters, abatement, and dietary guidance, can enhance these endpoints more cost-effectively than chelation therapy in borderline areas.
(3) Selective, safer chelation involves prioritizing ligands that target toxic metals with greater specificity than essential ones. It includes developing prodrugs activated specifically in target tissues and creating formulations that minimize hypersensitivity and gastrointestinal intolerance. Preclinical studies should compare these new agents directly against existing treatments like DMSA, DMPS, CaNa_2_EDTA, and BAL, paying close attention to mineral balance, microbiome impacts, and rebound effects early on. In early clinical trials, it is crucial to monitor both biomarkers and functional health outcomes, with efforts to include special populations such as children, pregnant women, and individuals with CKD whenever possible.
(4) Precision toxicology involves understanding the roles of the host and microbiome. Genetic polymorphisms such as those in AS3MT for arsenic methylation or in transporters for lead and cadmium, as well as microbiome composition, might account for individual differences in risk and treatment response. The focus of research should shift from mere associations to actionable insights: developing algorithms that combine genotype, metabolomics, diet, and environment to determine who needs chelation therapy, who would benefit most from prevention alone, and identifying the most effective agent and dosage. Clinical trials should evaluate genotype-guided chelation strategies against standard care, emphasizing patient-centered outcomes.
(5) Implementation science and equity are crucial. Even the most effective interventions will not succeed if they are not accessible where risk is highest. Cluster-randomized or stepped-wedge trials that combine environmental services (such as inspection, remediation, and filters) with clinical care should track metrics like time to source control, biomarker changes, adverse events, and QALYs, all broken down by social risk factors. Additionally, policies and payment systems should explore financing strategies, like utility subsidies, landlord incentives, or employer mandates, that support prevention efforts while reducing long-term health costs.
(6) Digital and decision-support tools, such as EHR-integrated pathways with default orders for essential tests, including speciation, and automatic notifications to public health, can speed up responses. Patient-facing apps can assist with fish selection, rice preparation, and filter upkeep. Research is needed to assess how well these tools are adopted, their usability, and their effects on outcomes and costs. Open-source toolkits would enable quick sharing across different resource settings.

Table 18 summarizes key future research and implementation priorities, highlighting exemplar study designs, primary endpoints, and the policy or clinical levers needed to translate evidence into practice.

## Figures and Tables

**Table 1:** Major natural sources, anthropogenic sources, environmental fate, and common exposure pathways of mercury, lead, cadmium, and arsenic.

Metal	Natural	Anthropogenic	Fate	Exposure	Refs.
**Hg**	Crust, volcanic systems, cinnabar	Coal, industry, wastewater, legacy products	Air transport, methylation, fish bioaccumulation	Seafood, vapor, occupational	[15]
**Pb**	Ores, galena	Mining, smelting, batteries	Soil/dust persistence, sediment binding	Dust, soil, water, food	[7]
**Cd**	Zn ores	Smelting, batteries, pigments, coal	Soil/crop accumulation, persistence	Food, inhalation, smoking	[16]
**As**	Crust, arsenic minerals	Smelting, coal, industry, groundwater	Groundwater/soil mobility, speciation-dependent toxicity	Water, rice/food, inhalation	[17]

**Table 2:** Mercury, lead, and cadmium species, deriving from human activities and their existence in biological systems through several consumptions.

Metal Compound	Sources	Refs.
***Elemental Mercury (Hg*** **^0^** ***)***	dental amalgams, fossil fuels	[24]
***Organic mercury (CH*** **_3_** ***Hg*** **^+^** ***)***	fish, poultry, pesticides	[24]
***Inorganic mercury (HgCl*** **_2_** ***)***	demethylation of organic mercury, oxidation of elemental mercury	[24]
***Lead***	mining, smelting, battery manufacturing, food, drinking water	[29]
***Cadmium***	production of nickel-Cd batteries, Cd-containing paint production, food (rice, potato)	[28]
***Inorganic arsenic (iAs; As(III)/As(V))***	groundwater/well water; rice and rice products; smelting/coal combustion; some traditional remedies	[17]

**Table 3:** Principal toxicokinetic features of mercury, lead, cadmium, and arsenic relevant to clinical assessment.

	Absorption	Distribution	Metabolism	Cause of Toxicity	Excretion	Refs.
**Elemental Mercury**	75–85% of vapor absorbed	BBB, kidney	Oxidized intracellularly to inorganic mercury by catalase and H_2_O_2_	Oxidation to inorganic mercury	Urine, feces, sweat, and saliva	[40]
**Organic Mercury**	95–100% in the intestinal tract; 100% of inhaled vapor	BBB, kidney	Slowly demethylated to inorganic mercury	Demethylation to inorganic mercury; binding to proteins	10% urine, 90% in bile, feces	[40]
**Inorganic Mercury**	7–15% of the ingested dose is absorbed	Kidney	Methylated by intestinal microflora	Binding to proteins	Urine, sweat, saliva, bile, feces	[40]
**Pb**	Gastrointestinal ingestion, inhalation	CNS, kidney	No metabolic transformation	Binding to proteins	Urine, a small portion in feces	[32]
**Cd**	Gastrointestinal ingestion, inhalation	Kidney, liver, skeleton	No metabolic transformation	Protein-binding	Urine, feces	[47]
**iAs**	Efficient GI absorption; inhalation (industry)	Systemic; liver and other tissues	Hepatic methylation → MMA/DMA	Thiol binding; enzyme inhibition; oxidative stress	Urine (major)	[9]

**Table 4:** Recommended reference levels for initiating chelation therapy in mercury, lead, and cadmium exposure.

Metal	Biomarker/Context	When Chelation May Be Considered	Notes	Refs.
**Mercury**	Blood, urine, or hair interpreted according to species and timing of exposure	Selected acute or symptomatic cases	Speciation and exposure history are essential; treatment depends on the form of mercury and the clinical presentation	[65]
**Lead**	Blood lead level in children	Generally considered at ≥45 µg/dL	Clinical judgment is still required; urgent treatment is needed for severe symptoms or very high levels	[31]
**Lead**	Adults	No single universal threshold	Decision depends on symptoms, exposure context, and specialist input	[66]
**Cadmium**	Blood or urine cadmium	Generally not recommended in routine practice	Benefit is limited and some chelators may worsen toxicity	[52]
**Arsenic (inorganic)**	Urine speciation and acute clinical presentation	Acute symptomatic inorganic arsenic poisoning	Early recognition, supportive care, and source control are critical	[62]

**Table 6:** Hard-Soft Acid-Base Theory [96].

Coordinating Groups			Metal Ions		
*Hard*	*Intermediate*	*Soft*	*Hard*	*Intermediate*	*Soft*
**H_2_O, OH^−^, RCOO^−^, NH_3_, RNH_2_, Cl^−^, F^−^, RO^−^**	C_6_H_5_NH_2_	RS^−^, RSH, R_2_S	Ca^2+^, Li^+^, Na^+^, K^+^, Mg^2+^, Al^3+^, Fe^3+^, Mn^2+^, Be^2+^, Sr^2+^ Ga^3+^, Cr^3+^, Sn^4+^, UO_2_^2+^, VO^2+^, (CH_3_)_2_Sn^2+^	Fe^2+^, Zn^2+^, Pb^2+^, Co^2+^, Ni^2+^, Cu^2+^, Bi^3+^, Sn^2+^, Sb^2+^	Ag^+^, Au^+^, Cu^+^, Pd^2+^, Pt^2+^, Pt^4+^, CH_3_Hg^+^, Hg^+^, Hg^2+^, Cd^2+^, As^3+^

**Abbreviations:** OH^−^, hydroxide; RCOO^−^, carboxylate; NH_3_, ammonia; RNH_2_, amine; Cl^−^, chloride; F^−^, fluoride; RO−, alkoxide; C_6_H_5_NH_2_, aniline; RS^−^, thiolate; RSH, thiol; R_2_S, thioether; Ca^2+^, calcium; Li+, lithium; Na^+^, sodium; K^+^, potassium; Mg^2+^, magnesium; Al^3+^, aluminum; Fe^3+^, ferric iron; Mn^2+^, manganese; Be^2+^, beryllium; Sr^2+^, strontium; Ga^3+^, gallium; Cr^3+^, chromium(III); Sn^4+^, tin(IV); UO_2_^2+^, uranyl; VO^2+^, vanadyl; (CH_3_)_2_Sn^2+^, dimethyltin; Fe^2+^, ferrous iron; Zn^2+^, zinc; Pb^2+^, lead; Co^2+^, cobalt; Ni^2+^, nickel; Cu^2+^, copper(II); Bi^3+^, bismuth; Sn^2+^, tin(II); Sb^2+^, antimony(II); Ag^+^, silver; Au^+^, gold; Cu^+^, copper(I); Pd^2+^, palladium; Pt^2+^, platinum(II); Pt^4+^, platinum(IV); CH_3_Hg^+^, methylmercury; Hg^+^, mercury(I); Hg^2+^, mercury(II); Cd^2+^, cadmium; As^3+^, arsenic(III).

**Table 8:** Clinical manifestations of primary heavy metals.

Metal	Acute Manifestations	Subacute/Chronic Manifestations	Hallmark Clinical Clues	Refs.
**Hg**	Pneumonitis (vapor)	Tremor, erethism, peripheral neuropathy	Neuropsychiatric changes; occupational exposure	[119]
**MeHg**	Rare acute GI effects	Cognitive decline, anemia, HTN, CKD	High-MeHg fish intake;	[120]
**Pb**	Vomiting, abdominal pain, encephalopathy	Cognitive impairment, HTN, CKD	Burton line	[121,122]
**Cd**	Acute pneumonitis and respiratory distress	Proteinuria, CKD, osteopenia, fractures	Smoking or occupational smelting exposure	[123]
**iAs/As**	Severe GI symptoms, dehydration, hypotension, QT interval prolongation,	Skin lesions, peripheral neuropathy, cancers	Raindrop hyperpigmentation	[124]

**Table 9:** Recent real-world heavy metal intoxication cases (2021–2024).

Year	Metal/Species	Exposure Scenario	Clinical Presentation/Key Findings	Main Clinical or Public-Health Lesson	Ref.
**2021**	Lead (Pb)	Frequent incense burning at home	Severe anemia, abdominal pain, bone pain, dyspnea, BLL 59.75 µg/dL; family cluster with elevated BLLs	Household practices may be overlooked sources; clustered cases suggest a shared source	[125]
**2021**	Lead (Pb)	Lead-adulterated opium	Adult poisoning outbreak; BLL and laboratory indices improved after treatment and exposure cessation	Substance adulteration can cause mass lead poisoning; source control is essential	[126]
**2022**	Arsenic (inorganic)	Pica with terracotta ingestion	Fatigue, constipation, paresthesia, urinary arsenic elevation, radiographic bowel opacities	Unusual ingestion behaviors should be considered in unexplained arsenic toxicity	[127]
**2022/2023**	Mercury	Contact with artisanal mining equipment	Mercury exposure associated with retained metallic material and vapor exposure	Mining-related exposure remains important; detailed occupational history is crucial	[128]
**2023**	Mercury chloride	Intentional ingestion	Rapid severe gastrointestinal/systemic toxicity; lethal blood mercury level	Inorganic mercury salts can be fatal and require urgent confirmation and support	[129]
**2024**	Lead (Pb)	Severe pediatric exposure	Refractory status epilepticus, severe microcytic anemia, blood lead >100 µg/dL	Lead toxicity should be considered in unexplained pediatric neurologic emergencies	[130]
**2024**	Cadmium (Cd)	Chronic exposure in polluted area	Renal dysfunction, osteomalacia, fractures, renal anemia; suspected itai-itai disease	Chronic cadmium toxicity still occurs in polluted regions with renal and skeletal effects	[131]

**Abbreviations:** BLL, blood lead level; GI, gastrointestinal.

**Table 10:** Differential diagnosis vs. metal-specific clues.

Clinical Problem	Common Differentials	Metal-Specific Clues	Refs.
**Neuropathy**	Diabetes mellitus, vitamin B12 deficiency, alcohol-related neuropathy	Exposure history; coexistence of neuropathy with renal dysfunction or skin changes may suggest toxic metal exposure	[132]
**Cognitive decline**	Depression, dementia, hypothyroidism	History of Pb exposure; associated anemia, neuropathy, or occupational/environmental risk factors	[133]
**Renal dysfunction**	NSAID nephropathy, autoimmune renal disease, diabetic kidney disease	Low-molecular-weight proteinuria suggests Cd nephrotoxicity	[134]
**Skin lesions**	Dermatitis, eczema, psoriasis	Hyperpigmentation and keratosis are characteristic clues for As exposure	[110]

**Table 11:** Clinically relevant toxic dose ranges and thresholds for Hg, Pb, Cd, and As.

Metal	Acute Threshold	Chronic Threshold of Concern	Preferred Matrix	Key Caveat	Refs.
**Hg**	Interpret according to species, symptoms, and timing; no single universal acute cutoff applies across all mercury forms	Blood, urine, or hair values should be interpreted by mercury species and exposure context	Blood, urine, hair	Species matters	[135]
**Pb**	Chelation usually at BLL ≥ 45 μg/dL children	CDC BLRV 3.5 μg/dL in children; no safe level identified	Venous blood	Use action thresholds	[136]
**Cd**	Acute toxicity mainly clinical	Urine Cd ~2–5 μg/g creatinine may indicate elevated body burden/renal risk	Urine	Reflects body burden	[61]
**As**	Acute toxicity is primarily clinical; urine speciation supports diagnosis	Interpret urine iAs/MMA/DMA, not total arsenic alone	Urine speciation	Avoid seafood before test	[137]

**Table 13:** Diagnostic biomarkers.

Metal	Preferred Biomarker	Matrix	Notes	Refs.
**Mercury (Hg)**	Total mercury with species-aware interpretation	Blood	Reflects recent methylmercury exposure	[140]
**Lead (Pb)**	Blood lead level	Whole blood	No safe pediatric threshold	[153]
**Cadmium (Cd)**	Urinary cadmium	Urine	Creatinine-corrected value preferred	[138]
**Arsenic (As)**	Speciated urinary arsenic	Urine	Avoid seafood before testing to reduce organic arsenic interference	[154]

**Table 14:** Management principles.

Setting	Primary Action	Key Tools	Clinical Goal	Refs.
**Home**	Lead and water hazard control	HEPA vacuuming, wet cleaning, certified filters, abatement	Reduce ongoing exposure	[156]
**Diet**	Reduce Hg/As/Cd intake without harming nutrition	Fish guidance, rice preparation, iron/calcium/zinc optimization	Lower intake while preserving diet quality	[167]
**Workplace**	Exposure prevention	Elimination/substitution, ventilation, PPE, hygiene, take-home prevention	Interrupt occupational exposure	[110]

**Table 16:** When biomarkers and outcomes diverge (or align).

Scenario	Biomarker Behavior	Outcome Signal	Clinical Implication	Refs.
**Dietary MeHg**	Hair/blood Hg ↓ after diet change	Symptoms often improve without chelation	Counsel diet; monitor to reinforce behavior	[167]
**Pediatric Pb (moderate, asymptomatic)**	BLL ↓ with DMSA	Neurodevelopment may not improve despite biomarker decline	Prioritize source control; selective chelation only when indicated	[169]
**Chronic Cd exposure/tubular injury risk**	Urine Cd reflects body burden; tubular markers (e.g., β2-microglobulin) change with injury;	Supportive care + exposure reduction slows progression; chelation lacks proven benefit and may add renal risk	No chelation; reduce exposure; monitor urine Cd + tubular markers; manage CKD/bone risk	[188]
**Acute iAs/Pb encephalopathy**	Rapid biomarker shifts with therapy	Outcomes improve with early chelation	Treat urgently; avoid delays	[170]

Abbreviations: ↓, decrease or decline in biomarker level after intervention or over time.

**Table 17:** Five-point summary for practice and policy.

Priority	What to Do	Why It Matters
**Source control**	Fix hazards, HEPA filtration, and workplace controls	Most significant health gain per dollar
**Right test**	Speciation, venous confirmation, Cr-corrected urine	Avoids misclassification and overtreatment
**Selective chelation**	Use when benefits > risks; clear stopping rules	Improves safety and value
**Equity**	Fund remediation for high-risk communities	Closing the gap where risk concentrates
**Measurement**	Track outcomes that matter to patients	Drives learning and accountability

**Abbreviations:** Cr-corrected, creatinine-corrected.

**Table 18:** Research agenda mapped to actionable endpoints.

Priority Domain	Exemplar Study Design	Primary Endpoint	Policy/Clinical Lever
**Rapid speciation**	Pragmatic diagnostic accuracy + outcome linkage	Correct classification; time-to-action	Reimburse POC tests
**Low-level endpoints**	Prospective cohort with standardized outcomes	MCID in cognition/renal/cardiometa-bolic endpoints	Update advisories/thresholds
**Safer chelation**	Phase 2/3 head-to-head vs. DMSA/DMPS/EDTA/BAL	Functional improvement + fewer AEs	Formulary and guideline adoption
**Precision algorithms**	Randomized, genotype-guided vs. usual care	Net benefit (symptoms, function, AEs)	Clinical decision support
**Bundled prevention**	Cluster/stepped-wedge trials	Time to source control; QALYs; equity gap closure	Housing/water/ occupational policy
**Digital tools**	Hybrid effectiveness-implementation	Adoption, adherence, outcomes, cost	Scale via open-source toolkits

## Data Availability

No new data were created or analyzed in this study. Data sharing is not applicable to this article.

## Abbreviations

ADME	Absorption, distribution, metabolism, and excretion
Ag	Silver
ALA	Alpha-lipoic acid
As	Arsenic
AS3MT	Arsenic (+3 oxidation state) methyltransferase
Au	Gold
BAL	British Anti-Lewisite (dimercaprol)
BLL	Blood lead level
CaNa_2_EDTA	Calcium disodium ethylenediaminetetraacetate
Cd	Cadmium
CKD	Chronic kidney disease
CNS	Central nervous system
Co	Cobalt
COOH	Carboxylic acid group
COO^−^	Carboxylate group
Cr	Creatinine
Cu	Copper
DHLA	Dihydrolipoic acid
DMA	Dimethylarsinic acid
DMPS	2,3-Dimercapto-1-propanesulfonic acid (unithiol)
DMSA	Dimercaptosuccinic acid (succimer)
DNA	Deoxyribonucleic acid
EDTA	Ethylenediaminetetraacetic acid
eGFR	Estimated glomerular filtration rate
Fe	Iron
FeAsS	Arsenopyrite (iron arsenic sulfide)
GI	Gastrointestinal
GSH	Glutathione
HEPA	High-efficiency particulate air
Hg	Mercury
Hg^0^	Elemental mercury
HgCl_2_	Mercuric chloride
HgS	Mercury sulfide
HgSe	Mercury selenide
iAs	Inorganic arsenic
IM	Intramuscular
IV	Intravenous
LA	Lipoic acid
MeHg	Methylmercury
MMA	Monomethylarsonic acid
Mn	Manganese
Ni	Nickel
O_3_	Ozone
Pb	Lead
PbO	Lead(II) oxide
PbO_2_	Lead(IV) oxide
PbS	Lead sulfide
pH	Potential of hydrogen (pH)
pKa	Acid dissociation constant (pKa)
PO	Oral administration (per os)
PPE	Personal protective equipment
QT	QT interval
ROS	Reactive oxygen species
Se	Selenium
SH	Thiol group
Sn	Tin
Zn	Zinc
ZnONPs	Zinc oxide nanoparticles
ZnS	Zinc sulfide
